# Increased Neutrophil Aging Contributes to T Cell Immune Suppression by PD-L1 and Arginase-1 in HIV-1 Treatment Naïve Patients

**DOI:** 10.3389/fimmu.2021.670616

**Published:** 2021-08-18

**Authors:** Kai Liu, Hui-Huang Huang, Tao Yang, Yan-Mei Jiao, Chao Zhang, Jin-Wen Song, Ji-Yuan Zhang, Chun-Bao Zhou, Jin-Hong Yuan, Wen-Jing Cao, Xiu-Ying Mu, Ming-Ju Zhou, Hua-Jie Li, Ming Shi, Ruonan Xu, Fu-Sheng Wang

**Affiliations:** ^1^Peking University 302 Clinical Medical School, Beijing, China; ^2^Department of Infectious Diseases, Fifth Medical Center of Chinese PLA General Hospital, National Clinical Research Center for Infectious Diseases, Beijing, China; ^3^Medical School of Chinese PLA, Beijing, China; ^4^Department of Clinical Medicine, Bengbu Medical College, Bengbu, China

**Keywords:** neutrophils, aging, HIV-1, immunosuppression, immunotherapy

## Abstract

Neutrophils are characterized by their heterogeneity. They fight against pathogens and are involved in tissue injury repair and immune system regulation. Neutrophils have an extremely short life span in the peripheral blood and undergo aging after being released from the bone marrow. The over-aggregation of aged neutrophils is associated with phenotypical and functional changes. Here, we aimed to investigate the dynamics of neutrophil aging and its relationship with T cell exhaustion in HIV-1 infection, as they are not well understood. In this study, we enrolled 23 treatment naïve (TN) patients, 23 individuals that had received antiretroviral therapy (ART), and 21 healthy controls (HC). In these cohorts, we measured the degree of neutrophil aging, and its possible correlation with T cell dysfunction. In TN patients, peripheral neutrophils showed a more distinct aging phenotype and were over-activated compared to those in ART-treated patients. The degree of neutrophil aging was positively correlated with HIV-1 RNA viral load and negatively correlated with CD4+ T cell count. Moreover, aged neutrophils had impaired reactive oxygen species (ROS) production after lipopolysaccharide (LPS) stimulation, and were characterized by increased PD-L1 and arginase-1 expression in a time-dependent manner. Aged neutrophils demonstrated an increased inhibition of IFN-γ and TNF-α secretion by CD8+ T cell compared to non-aged neutrophils. The inhibition effect could be partially reversed by blocking PD-L1 and arginase-1 *in vitro*, and LPS was identified as an important activator of neutrophil aging. These results provide evidence that dampening neutrophil aging may provide a novel approach to recover T cell dysfunction in patients with HIV-1 infection.

## Introduction

Immune aging is a significant feature of chronic HIV-1 infection and is associated with non-AIDS-related events. The occurrence of immune aging is closely related to the activation of the immune system (1, 2). Viral antigens and overexpressed pro-inflammatory cytokines are considered as the main activating factors that accelerate immune aging (2). Significant evidence points toward a close relationship and synergy among aging, immune activation, and immunosuppression (3). Neutrophils are the most abundant type of white blood cells in the peripheral blood, and their aging is not only associated with the circadian rhythm but also affected by exogenous activators (4). Currently, the complete understanding of neutrophil aging and its role in T cell exhaustion and involvement in the complete immune reconstitution of patients with HIV-1 infection are lacking (5).

Blood polymorphonuclear neutrophils are the first-line immunocytes to arrive at the site of infection and fight against pathogens (6, 7). During HIV-1 infection, neutrophils defend the body by secreting neutrophil extracellular traps (NETs), myeloperoxidase, α-defensin, and other factors (8). Simultaneously, neutrophils have a characteristic heterogeneity, which mediates immunosuppression *via* PD-L1, arginase-1, and reactive oxygen species (ROS) in many diseases including cancer and systemic lupus erythematosus (9–11).

Under normal physiological conditions, the life span of a neutrophil is less than approximately 24 h without any activation (12). Approximately 100 billion neutrophils are released into the blood from bone marrow daily, and once released, neutrophil aging occurs, which is characterized by increased CXCR4 and decreased CD62L expression. Aged neutrophils are returned to the bone marrow upon interaction *via* CXCL12, and cleared by resident macrophages (13–15).

Neutrophil aging is associated with gut microbiota and the circadian rhythm. Aged neutrophils are characterized by structural changes, including enlarged cell size, increased nuclear lobules, and altered phenotype (16). Germ-free status or microbiota depletion dampens aging, while LPS and fecal transplantation restore it (13). Aged neutrophils are more likely to migrate to infection sites and exhibit strong phagocytic ability by releasing ROS, NETs, and other factors. Previous studies in human ischemic stroke showed that aged neutrophils with a CD62L^lo^CXCR4^+^ phenotype are elevated, and their levels are associated with disease severity (17). Over-aggregation of aged neutrophils induces thrombotic inflammation (16, 18, 19). Furthermore, neutrophils are characterized by immune activation and high PD-L1 expression in patients with gastric cancer; therefore, the immunosuppression may be introduced by neutrophils (3).

The aging status of neutrophils and its relationship with T cell exhaustion are largely unknown during HIV-1 infection. Therefore, here, we aimed to investigate this further by observing neutrophil aging in both patients that had received antiretroviral therapy (ART) and treatment naïve (TN) patients with HIV-1 infection.

## Materials and Methods

### Study Participants

Patients were enrolled from the Fifth Medical Center, General Hospital of PLA, Beijing, China, between January 2020 and December 2020. A total of 23 TN (with unrestricted CD4+ T cell counts) patients diagnosed with HIV-1 infection and 23 ART-treated patients with HIV-1 viral load below the limit of detection for at least 24 months were enrolled. A total of 21 healthy donors were enrolled as controls. Patients with the following features were excluded: 1) acute bacterial, as well as HBV and HCV infection; 2) tumor and other serious organ diseases unrelated to HIV-1 infection; and 3) long-term immunosuppressive therapy. The age, gender, viral load and CD4/CD8 count of all of the patients are listed in **Table 1**.

**Table 1:** Clinical characteristics of participants^a^.

Patients	HC (n = 21)	TN (n = 23)	ART (n = 23)
Age (y)	28 (24–39)	29 (21–52)	30 (21–57)
Gender (female/male)	9/12	1/22	0/23
Viral Load (log10/ml)	NA	3.87 (2.15–4.97)	<LDL
CD4^+^ T cell Count (cells/μl)	796 (427–1,013)	318 (4–428)	565 (433–947)
CD8^+^ T cell Count (cells/μl)	642 (272–1,744)	1,098 (278–1,345)	699 (318–1,229)
CD4/CD8 ratio	1.06 (0.52–1.91)	0.26 (0.01–1.13)	0.8 (0.48–1.49)

HC, healthy controls; TN, treatment-naïve HIV-1-infected patients; ART, HIV-1-infected patients with long-term ART; LDL, the low detection limit.

a All items, except gender, are median values with range.

Heparinized and EDTA anti-coagulated peripheral venous blood samples were collected aseptically from patients and healthy controls (HCs). All blood samples were collected between 8 and 9 AM. Phenotype and function tests were performed within 2 h after blood samples were collected.

This study was approved by the institutional review boards of the Fifth Medical Center of Chinese PLA General Hospital. Study subjects provided written informed consent in line with the Declaration of Helsinki.

### Neutrophil Isolation and Flow Cytometry Analysis

Peripheral blood mononuclear cells (PBMCs) were isolated *via* centrifugation on a Ficoll–Paque PLUS gradient (GE Healthcare, Uppsala, Sweden). Neutrophils were further isolated after dextran sedimentation and hypotonic lysis as previously described (20).

Fresh EDTA anti-coagulated peripheral blood (100 μl) was incubated with antibodies for 30 min in the dark. The following antibodies were used: Anti-CD66b-PE-cy7, anti-TLR2-FITC, anti-TLR4-APC, anti-CXCR1-APC, anti-CXCR2-FITC, anti-C5aR-APC, anti-CD64-PE, anti-CD177-APC, anti-CD62L-FITC, anti-CD11b-PE, anti-CD49d- FITC, anti-C5L2-PE, anti-CD3-BV421 and anti-PD-1-PE were obtained from Biolegend (San Diego, California, USA); anti-CD4-BV421, anti-CD8-APC-cy7, anti-CD38-FITC, and anti-HLA-DR-PerCP were purchased from BD Biosciences (Franklin Lakes, New Jersey, USA); anti-PD-L1-PE were bought from eBioscience (San Diego, California, USA). Isotype-matching antibodies were used as negative controls. After lysing the red blood cells, the remaining cells were washed and fixed for flow cytometry analysis.

### Aging Score

Aging score was defined as described in a previous study (21). In brief, aging scores were determined as the weighted sum of z-scores according to age-related phenotypes. Phenotype weights were set to 1 or −1 according to the changes accompanied by cell aging. The phenotype markers including CXCR4, CD62L, CXCR2, CD49d, and CD11b were selected as previous recommendation (22).

### Oxidative Burst Assay

The oxidative burst of neutrophils was determined according to the manufacturer’s instructions. Briefly, 100 μl of heparinized whole blood was pretreated by incubating in an ice bath for 10 min. LPS (Sigma Aldrich, St. Louis, MO, USA) at a final concentration of 100 ng/ml or the same volume of PBS was added. The burst assay samples were incubated for 10 min at 37.0°C in a water bath. Dihydrorhodamine (DHR) 123 (AAT Bioquest, Sunnyvale, CA, USA) was added and the sample was mixed for another 10 min. After staining, erythrocytes were lysed, and the remaining cells were fixed and then collected for flow cytometry analysis.

### Neutrophil Aging Induction

The heparinized whole blood was mixed with equal volumes of RPMI-1640, incubated at 37°C for 4, 8, 12, 24, and 48 h, and then stained with anti-PD-L1-PE, anti-CXCR4-APC, anti-CD62L-FITC, and anti-CD66b-PE-cy7, isotype-matching antibodies were used as negative controls. Levels of arginase-1 in the supernatant were detected using an enzyme-linked immunosorbent assay (ELISA).

Isolated neutrophils were incubated with LPS (10 ng/ml) (Sigma Aldrich, St. Louis, MO, USA) and GM-CSF (100 ng/ml) (Huabei Pharmaceutical Co. Ltd. Shijiazhuang, China) separately or coordinately for 12 h *in vitro*. Staining analysis for anti-PD-L1-PE, anti-CXCR4-APC, anti-CD62L-FITC, and anti-CD66b-PE-cy7 were conducted. Cells were fixed in 0.5% formaldehyde and analyzed using flow cytometry on a BD Canto II flow cytometer (BD Biosciences).

### Neutrophil and T Cell Co-Culture *In Vitro*

Freshly isolated neutrophils and aged neutrophils derived from the *in vitro* culture for 24 h were separately co-cultured with PBMCs at a 3:1 ratio. Anti-CD3 (1 μg/ml)/anti-CD28 (1 μg/ml) was added. For staining intracellular markers, PBMCs were permeabilized utilizing the Permeabilization/Fixation Kit from eBioscience (San Diego, California, USA). Samples were incubated with anti-IFN-γ-PE-cy7 and anti-TNF-α-PE antibodies. Cells were fixed in 0.5% formaldehyde and analyzed using flow cytometry on a BD Canto flow cytometer (BD Biosciences).

For blocking assay, isolated neutrophils were cultured with or without a neutralizing antibody against human PD-L1 (10 μg/ml) (Ebioscience, San Diego, California, USA) and arginase-1 inhibitor nor-NOHA (250 μM) (Biovision, Milpitas, CA, USA) for 1 h at 37°C, the corresponding IgG control was also used. Samples were co-cultured with autologous PBMCs at a 3:1 ratio containing anti-CD3 (1 μg/ml) and anti-CD28 (1 μg/ml) antibodies for another 12 h. The cells were then harvested for intracellular cytokine staining. Fixed cells were further analyzed using FACS Canto and FlowJo (Tristar, San Carlos, CA) software.

### Soluble Marker Quantification

Arginase-1 (Hycult, Uden, The Netherlands) levels were determined using ELISA according to the manufacturers’ instructions. Serum levels of ten different cytokines (IL-1β, IL-6, IL-8, IFN-γ, TNF-β, IL-17A, IL-18, neutrophil gelatinase-associated lipocalin (NGAL), GM-CSF, and G-CSF) were determined *via* flow cytometry using a QBPlex Human custom 10-plex Kit (Quantobio, Beijing, China).

### Statistical Analysis

Statistical analysis was performed using GraphPad Prism 7.0 (GraphPad Software, San Diego, CA, USA) and SPSS (Version 24, Chicago, SPSS Inc). Continuous and normally distributed data were analyzed using a t-test or one-way analysis of variance. Mann–Whitney U-test was used for non-normally distributed continuous data. Correlations were determined using Pearson analysis. All data are expressed as the mean ± standard error of the mean. *p <* 0.05 was considered to indicate statistical significance.

## Results

### Neutrophil Aging Is Increased in HIV-Infected Patients and Is Positively Correlated With HIV RNA and T Cell Activation

**Figure 1:**
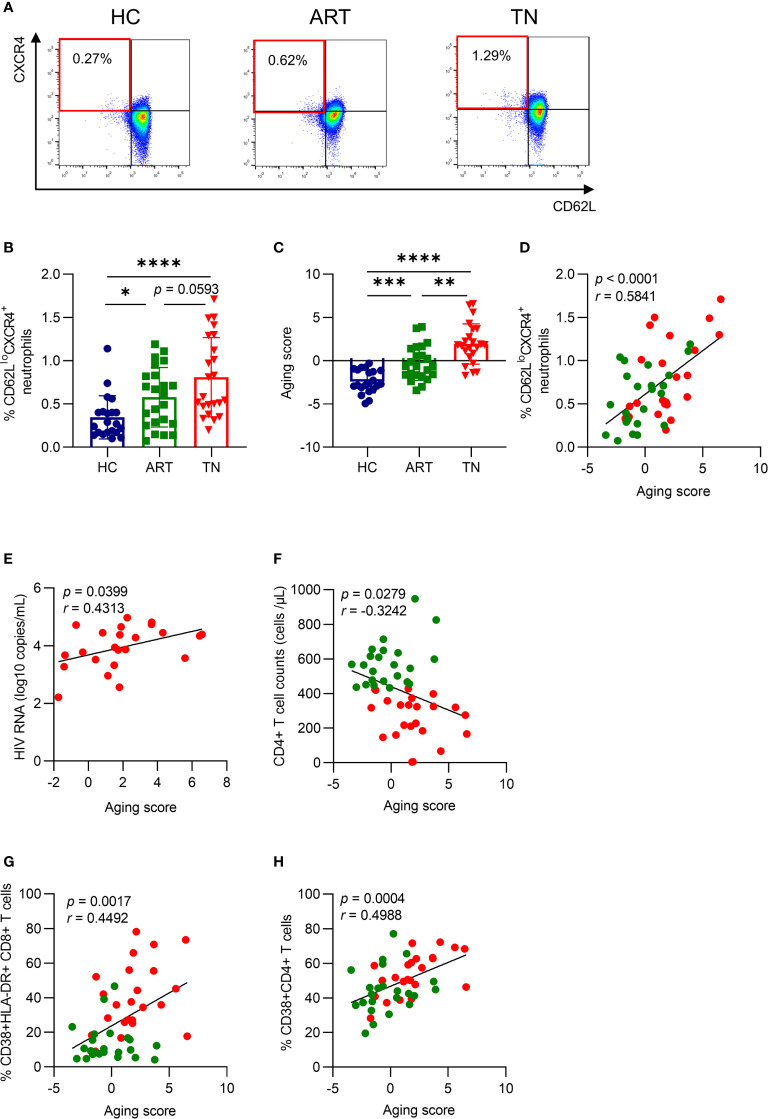
Neutrophil aging is increased in HIV-infected patients and is positively correlated with HIV RNA and T cell activation. **(A)** Representative gating diagram for aged neutrophils in healthy controls (HCs), antiretroviral therapy (ART)-treated and ART treatment naïve (TN) patients. Aged neutrophils were defined as CD66b^+^CD62L^lo^CXCR4^+^. **(B)** Differences in CD62L^lo^CXCR4^+^ expression on neutrophils among groups. **(C)** Differences in the aging score of neutrophils among groups. **(D)** The relationship between the aging score and the percentage of CD62L^lo^CXCR4^+^ neutrophils. For statistical analyses, Mann–Whitney U-test was performed. *p < 0.05, **p < 0.01, ***p < 0.001, ****p < 0.0001. **(E)** The correlation of the neutrophil aging score with viral load in TN patients. **(F)** The correlation of neutrophil aging score with CD4^+^ T cell count in HIV-1-infected patients. **(G, H)** The relationship between the aging score and the percentage of CD38^+^HLA^-^DR^+^CD8^+^ T cells **(G)** and CD38^+^CD4^+^ T cells **(H)**. Statistical analyses were performed using Pearson correlation tests.

To identify the degree of neutrophil aging in HIV-1-infected patients, we measured the percentage of CD62L^lo^CXCR4^+^ neutrophils, as previously described (13, 16), which was increased in TN patients and was not fully recovered under efficient ART treatment (**Figures 1A, B** **)**. In addition, the aging score was determined simultaneously by calculating the weighted sum of *z*-scores according to age-related markers (22), which included CXCR4, CD62L, CXCR2, CD49d, and CD11b, and the mean fluorescence intensity (MFI) of each marker was shown (**Supplementary Figures 1A–E**). The MFI of CXCR4 increased, while that of CD62L and CXCR2 both decreased in TN patients. Although the MFI of CD49d and CD11b showed no significant difference within different groups, all five markers showed a correlation with the aging score (**Supplementary Figure 1F**). The aging score increased in TN patients (**Figure 1C**) and was coordinated with the percentage of CD62L^lo^CXCR4^+^ neutrophils (*r =* 0.5841, *p <*0.0001) (**Figure 1D**). Interestingly, the aging score of neutrophils was positively correlated with HIV-1 RNA viral load (*r =* 0.4313, *p =* 0.0399) and negatively correlated with CD4+ T cell counts (*r =* −0.3242, *p =* 0.0279) at enrollment (**Figures 1E, F** **)**. Meanwhile, the aging score was positively correlated with the percentage of CD38+HLA-DR+CD8+ T cells (*r =* 0.4492, *p =* 0.0017) and CD38+CD4+ T cells (*r =* 0.4988, *p =* 0.0004) (**Figures 1G, H** **)**. This indicated that aged neutrophils were closely associated with HIV viral load, as well as CD4+ T cell count and activation. Still, these results did not fully elucidate the outcome and the reason for neutrophil aging.

### Aged Neutrophils From HIV-Infected Patients Have Impaired ROS Production After LPS Stimulation and Exhibit Over-Activation

**Figure 2:**
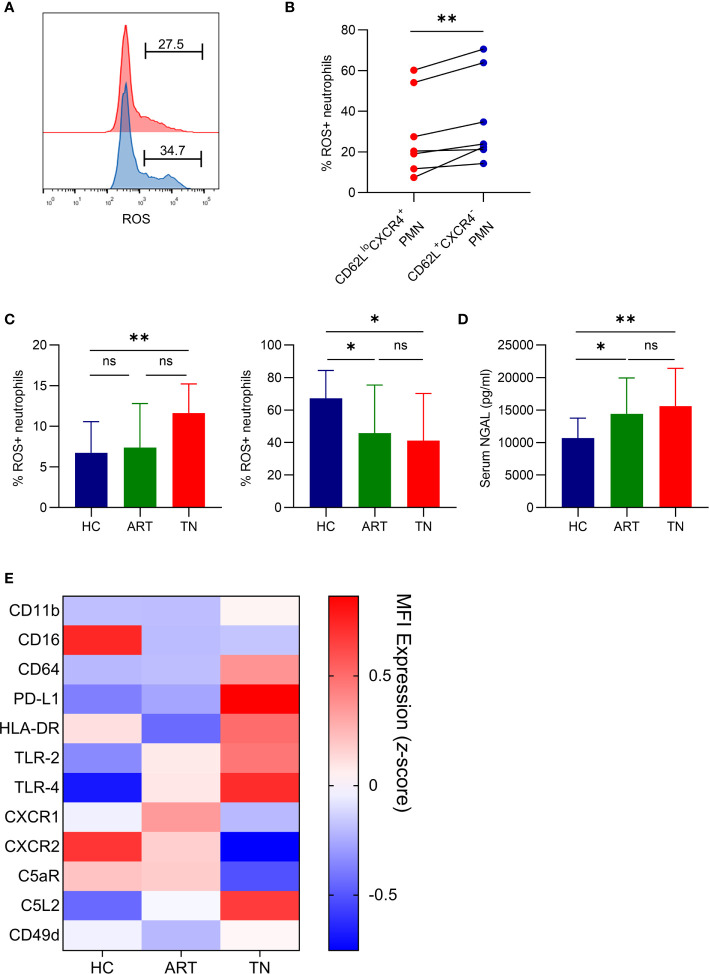
Aged neutrophils from HIV-infected patients have impaired ROS production after LPS stimulation and exhibit over-activation. **(A, B)** Representative flow cytometric plots gated for CD66b^+^CD62L^lo^CXCR4^+^ROS^+^ (red) and CD66b^+^CD62L^+^CXCR4^-^ROS^+^ (blue) neutrophils **(A)**, and statistical data **(B)** for ROS production by CD62L^lo^CXCR4^+^ and CD62L^+^CXCR4^-^ neutrophils after LPS activation. **(C)** Spontaneous (left) and LPS-induced (right) production of ROS by neutrophils detected using FACS analysis. **(D)** Plasma levels of NGAL in healthy controls (HCs) and HIV-1-infected patients. For statistical analyses, Mann–Whitney U-test or t-test was performed. *p < 0.05, **p < 0.01, ns, not significant. **(E)** Comparison of phenotypical features of neutrophils in HCs, antiretroviral therapy (ART)-treated and ART treatment naïve (TN) patients. The expression of CD62L, CD11b, CD16, CD64, PD-L1, HLA-DR, TLR-2, TLR-4, CXCR1, CXCR2, C5aR, C5L2, CD49d, and CXCR4 was detected using flow cytometry, and the data are shown on a heatmap. Each color represents the MFI of the indicated marker (*z*-score). PMN, polymorphonuclear neutrophil.

Although our data revealed that neutrophil aging increased in HIV-1-infected patients, we did not fully understand the function of the aged neutrophils. ROS production was separately monitored in aged (CD62L^lo^CXCR4^+^) and non-aged (CD62L^+^CXCR4^-^) neutrophils. Aged neutrophils had a lower ROS production after LPS stimulation when compared with non-aged neutrophils (**Figures 2A, B** **)**. In TN patients, the spontaneous production of ROS in neutrophils was higher than that in HCs, and LPS-induced ROS production was lower (**Figure 2C**). Therefore, the neutrophils in TN patients might be dysfunctional and have impaired response to LPS stimulation as a result of aging. Neutrophil dysfunction in TN patients was also identified by an increased concentration of NGAL, a marker of neutrophil degranulation (**Figure 2D**). Notably, after screening the phenotype of neutrophils, we found that neutrophils in TN patients exhibited an over-activated phenotype, which was characterized by elevated CXCR4, PD-L1, TLR-2, TLR-4, and C5L2 expression, as well as decreased CD16, CXCR2, C5aR, and CD62L expression (**Figure 2E**). These results indicated that neutrophils in TN patients were over-activated and had impaired ROS production after LPS stimulation.

### Aged Neutrophils Have High PD-L1 and Arginase-1 Expression in TN Patients

**Figure 3:**
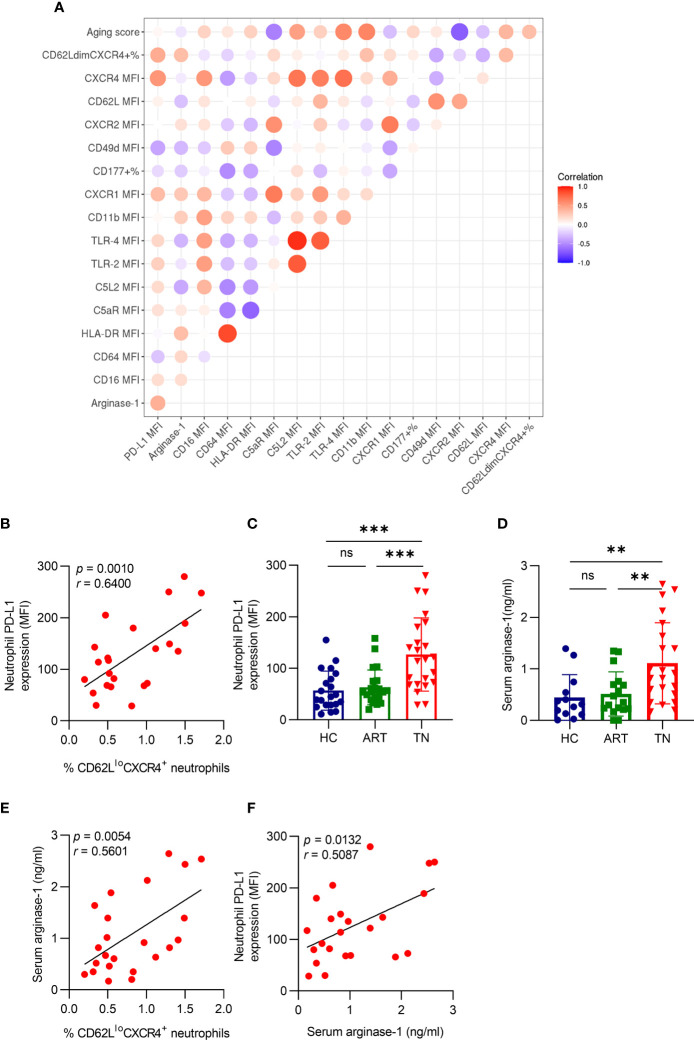
Aged neutrophils have high PD-L1 and arginase-1 expression in TN patients. **(A)** The Spearman’s correlation of neutrophil characters presented in a heatmap. The colored scale bar ranging from blue to red (−1 to 1) corresponds to negative and positive correlations, respectively. The size of the circle represents the *p*-value. **(B)** Correlation between CD62L^lo^CXCR4^+^ and PD-L1 expression on neutrophils in HIV-1-infected patients. **(C)** Comparison of PD-L1 expression on neutrophils in HIV-1 patients. **(D)** Comparison of the plasma-derived arginase-1 in HIV-1-infected patients. **(E)** Correlation between CD62L^lo^CXCR4^+^ and plasma arginase-1 levels in TN patients. **(F)** Correlation between PD-L1 expression on neutrophils with arginase-1 in HIV-1 TN patients. For statistical analyses, Mann–Whitney U-test was performed. Correlations were calculated using Pearson’s correlations. **p < 0.01, ***p < 0.001, ns, not significant.

To analyze the relationship between aging and other neutrophil phenotypes, we used multivariate correlation analysis and found that the percentage of CD62L^lo^CXCR4^+^ neutrophils was positively correlated with PD-L1 expression (*r =* 0.6400, *p =* 0.0010) (**Figures 3A, B** **)**. In addition, we detected PD-L1 expression on neutrophils in different groups and found that it was higher in TN patients, but no significant difference was observed between HC and ART treatment groups (**Figure 3C**). The arginase-1 expression consistently increased in TN patients (**Figure 3D**), and was positively correlated with the percentage of CD62L^lo^CXCR4^+^ neutrophils (*r =* 0.5601, *p =* 0.0054) (**Figure 3E**) and PD-L1 expression (*r =* 0.5087, *p =* 0.0132) (**Figure 3F**). These results indicated that PD-L1 and arginase-1 expression both increased in TN patients, and were associated with the severity of neutrophil aging.

### PD-L1 and Arginase-1 Expression Is Accompanied by Neutrophil Aging *In Vitro*

**Figure 4:**
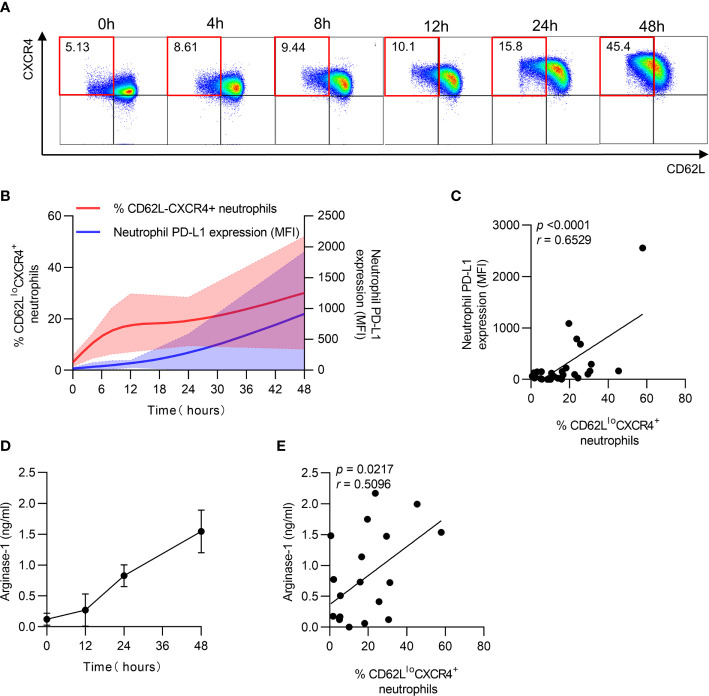
PD-L1 and arginase-1 expression is accompanied by neutrophil aging *in vitro*. **(A)** Percentage of CD62L^lo^CXCR4^+^ neutrophils increased after *in vitro* culturing. Neutrophils were cultured *in vitro* for 48 h, and the expression of CD62L and CXCR4 was detected using FACS. **(B)** Dynamics of CD62L^lo^CXCR4^+^ neutrophils and PD-L1 expression after *in vitro* culturing. **(C)** Relationship between the percentage of CD62L^lo^CXCR4^+^ neutrophils and PD-L1 MFI expression. **(D)** Dynamic change of arginase-1 secretion after neutrophils was cultured *in vitro*. **(E)** Correlation between the proportion of CD62L^lo^CXCR4^+^ neutrophils and arginase-1 production. For statistical analyses, Mann–Whitney U-test was performed. Correlations were calculated using Pearson’s correlations.

After identifying the relationship between PD-L1 and arginase-1 expression and neutrophil aging in patients, the dynamics of PD-L1 and arginase-1 expression on neutrophils were measured *in vitro*. The percentage of CD62L^lo^CXCR4^+^ neutrophils was increased, accompanied by increased PD-L1 expression after culturing for long periods (**Figures 4A, B** **)**. Furthermore, the aged neutrophils had higher PD-L1 expression than the non-aged ones (**Supplementary Figures 2A, B** **)**, and PD-LI expression increased along with the percentage of CD62L^lo^CXCR4^+^ neutrophils during the extended culture time (**Figure 4C**). In addition, arginase-1 expression increased with time and was positively correlated with the CD62L^lo^CXCR4^+^ phenotype (**Figures 4D, E** **)**. These findings implied that neutrophil aging was accompanied by increased PD-L1 and arginase-1 expression.

### Aged Neutrophils Play an Immunosuppressive Role Through PD-L1 and Arginase-1

**Figure 5:**
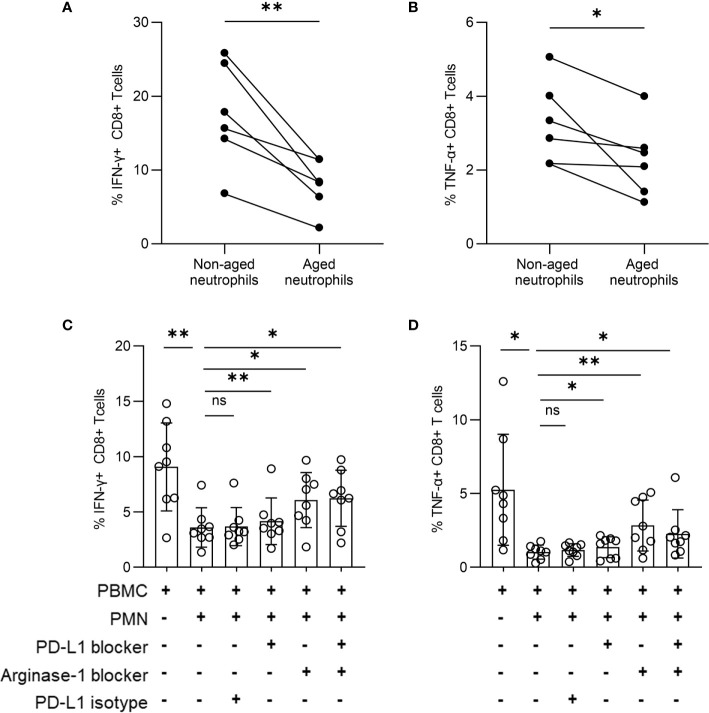
Aged neutrophils play an immunosuppressive function through PD-L1 and arginase-1. **(A, B)** PBMCs were co-cultured with neutrophils derived from an *in vitro* culture for 0 h, 24 h at a 1:3 ratio. Statistical analysis of CD8^+^ T cells secreting IFN-γ **(A)** and TNF-α **(B)**. **(C, D)** PBMCs were stimulated by anti-CD3 (1 μg/ml) and anti-CD28 (1 μg/ml) antibodies with or without neutrophils from TN patients at a 1:3 ratio for 12 h in the presence or absence of a specific PD-L1-blocking antibody or arginase-1 inhibitor (nor‐NOHA). Cumulative IFN-γ **(C)** and TNF-α **(D)** expression was measured *via* intracellular cytokine staining of CD8^+^ T cells. For statistical analyses, Mann–Whitney U-test was performed. *p < 0.05, **p < 0.01, ns, not significant.

To identify the effect of aged neutrophils on T cells, we cultured PBMCs and neutrophils together, and found the percentage of IFN-γ+ CD8+ along with TNF-α+ CD8+ T cells was decreased after co-culturing with aged neutrophils for 24 h (**Figures 5A, B** **)**, while no significant change occurred when PBMCs were cultured alone. The results can be interpreted that aged neutrophils had an increased immunosuppression effect on T cells. The addition of a blocking assay was performed by culturing PBMCs and neutrophils together with anti-PD-L1 antibody and arginase-1 inhibitor. After 12 h of culture, the expression of IFN-γ and TNF-α in CD8+ T cells was measured. We found that the addition of neutrophils induced a significant decrease of IFN-γ and TNF-α expression in CD8+ T cells, and the inhibition effect was partially reversed by PD-LI and arginase-1 blocking (**Figures 5C, D** **)**. Meanwhile, PD-L1 and arginase-1 inhibition had no significant effect on IFN-γ and TNF-α secretion in CD8+ T cells *in vitro* (**Supplementary Figures 3A, B** **)**. We hypothesized that both PD-L1 and arginase-1 derived from aged neutrophils were involved in the immunosuppression of CD8+ T lymphocytes.

### Neutrophil Aging Is Associated With Pro-Inflammatory Cytokines

**Figure 6:**
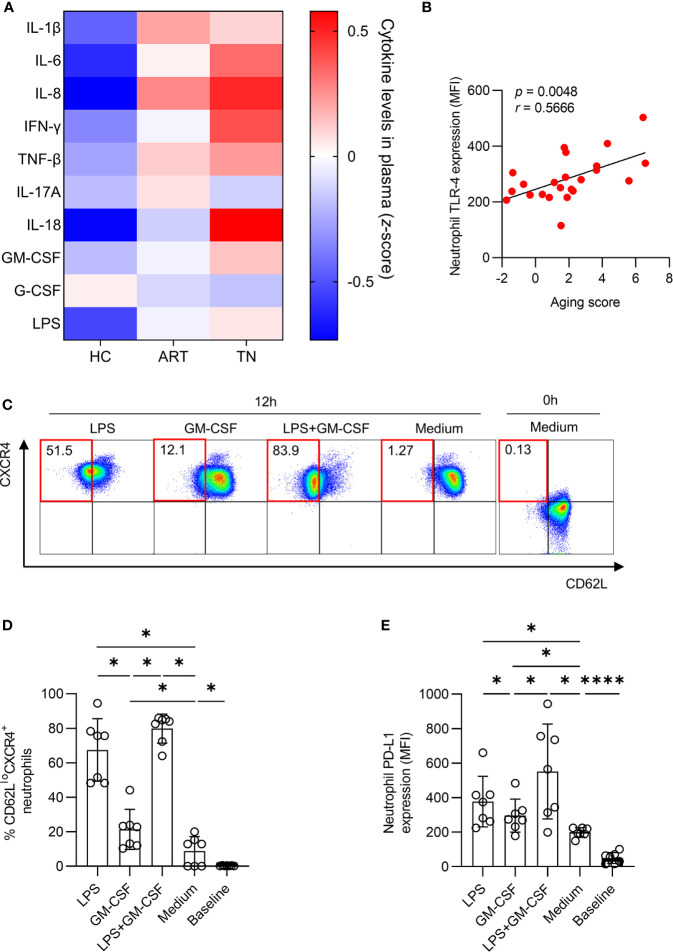
LPS accelerates neutrophil aging. **(A)** Comparison of plasma cytokines among healthy controls (HCs), antiretroviral therapy (ART)-treated and ART treatment naïve (TN) patients. The expression of IL-1β, IL-6, IL-8, IFN-γ, TNF-β, IL-17A, IL-18, GM-CSF, G-CSF, and LPS was detected, and the data are shown on a heatmap. The color in each cell represents the level of indicated cytokine relative to all samples (*z*-score). **(B)** Correlation of aging score of neutrophils with TLR-4 expression in TN patients. **(C–E)** LPS and GM-CSF induced PD-L1 expression and increased the percentage of CD62L^lo^CXCR4^+^ neutrophils. Purified neutrophils were incubated with or without LPS or GM-CSF for 12 h. Representative diagram **(C)**, the percentage of CD62L^lo^CXCR4^+^ neutrophils **(D)** and PD-L1 expression **(E)** among total neutrophils were shown. For statistical analyses, Mann–Whitney U-test was performed. Correlations were calculated using Pearson’s correlations. *p < 0.05, ****p < 0.0001.

To identify the inflammatory mediators that may be responsible for mediating neutrophil aging, we screened IL- 1β, IL-6, IL-8, IFN-γ, IL-18, TNF-β, IL-17A, GM-CSF, G-CSF, and LPS levels in the plasma (**Supplementary Figures 4A–J**). We observed that IL-1β, IL-6, IL-8, IFN-γ, IL-18, and LPS levels were all significantly elevated in TN patients compared to those in HCs (**Figure 6A**). The expression of TLR-4, a receptor of LPS, was positively correlated with the aging score of neutrophils (*r =* 0.5666, *p =* 0.0048) (**Figure 6B**). Next, we investigated the influence of LPS on neutrophil aging, the data showed that LPS had a stronger capacity to improve the percentage of CD62L^lo^CXCR4^+^ and PD-L1 expression on neutrophils than GM-CSF. Furthermore, LPS and GM-CSF had a synergistic effect *in vitro* (**Figures 6C–E**).

## Discussion

Neutrophil aging is an important physiological phenomenon for the preservation of immunological homeostasis, but under pathological conditions, over-aging may contribute to increased tissue pathogenesis and immune disorders (23). CD62L^lo^CXCR4^+^ subsets, defined as aged neutrophils, have stronger antibacterial activities in acute inflammation (15), but their role in chronic HIV-1 infection are not well understood. In this study, we demonstrated that aging neutrophils accumulate in TN patients with HIV-1 infection and exhibit immunosuppression partially through PD-L1 and arginase-1.

Neutrophils are important immune system guards during HIV-1 infection (8). High neutrophil counts before infection are associated with a low risk of sexually transmitted HIV-1 infection, and low neutrophil counts in mothers are associated with a high risk of perinatal infection (24). Emerging evidence has shown that neutrophils are phenotypically and functionally heterogeneous (24). Under normal physiological conditions, neutrophil heterogeneity may arise from aging and is associated with functional changes (25).

Well-known markers, CD62L and CXCR4, were used to evaluate neutrophil aging, where lower CD62L and higher CXCR4 expression indicated increased neutrophil aging. In our study, we found that the percentage of CD62L^lo^CXCR4^+^ cells was significantly expanded in the peripheral blood of TN patients with HIV. As another validation, the aging score, including CD62L, CXCR4, CXCR2, CD49d, and CD11b status, was introduced and applied to evaluate the severity of neutrophil aging among HIV-1-infected patients. Both methods showed good consistency. Via further analysis, we found that the aging score was negatively correlated with CD4+ T cell counts, and positively correlated with HIV-1 RNA viral load and T cell activation. These results indicated that the virus may be an important factor in neutrophil aging, which happens in coordination with T cell activation. In infection-induced kidney injury animal models, aged neutrophils have a higher capacity to fight against fungal infections than non-aged ones (16). In our study, we found that the aged neutrophils have impaired ROS production after LPS stimulation. In addition, spontaneous ROS and NGAL productions were increased in TN patients, and the total number of neutrophils in TN patients showed an over-activation phenotype with high expression of TLR-2, TLR-4, C5L2, CD16 and low expression of CXCR2 and C5aR. This indicated that the persisting activation and paralysis of neutrophils may exist *in vivo*, and ultimately weaken the proper immune function of these cells.

In peripheral blood, neutrophils are terminally differentiated and have a short lifespan (23). Neutrophil aging occurs in the peripheral blood after being released from bone marrow, with increased CXCR4 and decreased CD62L expression. Neutrophil turnover is not only associated with aging but also with changes in immune-phenotype and function. In a previous study, neutrophils with high PD-LI expression showed obvious immunosuppression of CD8+ T cells in HIV-infected patients (11). In our study, we found that the expression of both PD-L1 and arginase-1 significantly increased in TN patients and were positively correlated to the percentage of CD62L^lo^CXCR4^+^ neutrophils and aging score. This indicated that neutrophil phenotype and function both change as aging progresses, and aged neutrophils may have an immunosuppressive effect on T cells.

*In vitro*, PD-L1 and arginase-1 expression increased with aging, and aged neutrophils even had a higher capacity to inhibit IFN-γ and TNF-α production in CD8+ T cells than non-aged cells. Although the arginase-1 had a more efficient inhibition effect than PD-LI, it still needs further investigation whether the slight reversing effect of the PD-LI-blocking antibody used in our study was due to the lack of PD-1/PD-L1 or not. In addition, arginase-1 and PD-LI blocking only partially reversed the immunosuppression of CD8+ T cells by neutrophils, other immunomodulation pathways may further be proposed.

During HIV-1 infection, microbial translocation caused by gastrointestinal mucosal damage is a major driver for HIV-1-related events (26), and LPS mediates the adaptive immune and innate immune activation (27–29). In an antibiotic-treated mice model, neutrophil aging was controlled by LPS and/or microbiota *via* Toll-like receptor signaling. Depletion of the microbiota significantly reduces the number of circulating aged neutrophils and dramatically alleviates the pathogenesis and inflammation-related organ damage (13).

In our study, we found that LPS increased in TN patients, and the expression of TLR-4 on neutrophils was positively correlated with the aging score. LPS, as an activator, showed a stronger capacity to improve the percentage of CD62L^lo^CXCR4^+^ neutrophils than GM-CSF. The synergistic effects of LPS and GM-CSF indicated complex interactions between the inflammatory environment and neutrophil aging. Other inflammatory cytokines including IL-1β, IL-6, IL-8, IFN-γ, and IL-18 may also be involved but were not identified in our study. In addition, aged neutrophils in the peripheral blood were mainly eliminated by macrophages; therefore, dysfunction of macrophages may be another important factor contributing to the accumulation of aged neutrophils in patients with HIV infection, which warrants further investigation.

There are some limitations in our study. The progression of neutrophil aging may be associated with a longer lifespan, inflammation, decreased phagocytosis by macrophages, and other factors; however, we only identified neutrophil aging in TN patients with HIV-1 infection, and the associated mechanisms of aging were not fully confirmed. Notably, the relationship among aging, activation, and immunosuppression cannot be fully differentiated by our current data.

**Figure 7:**
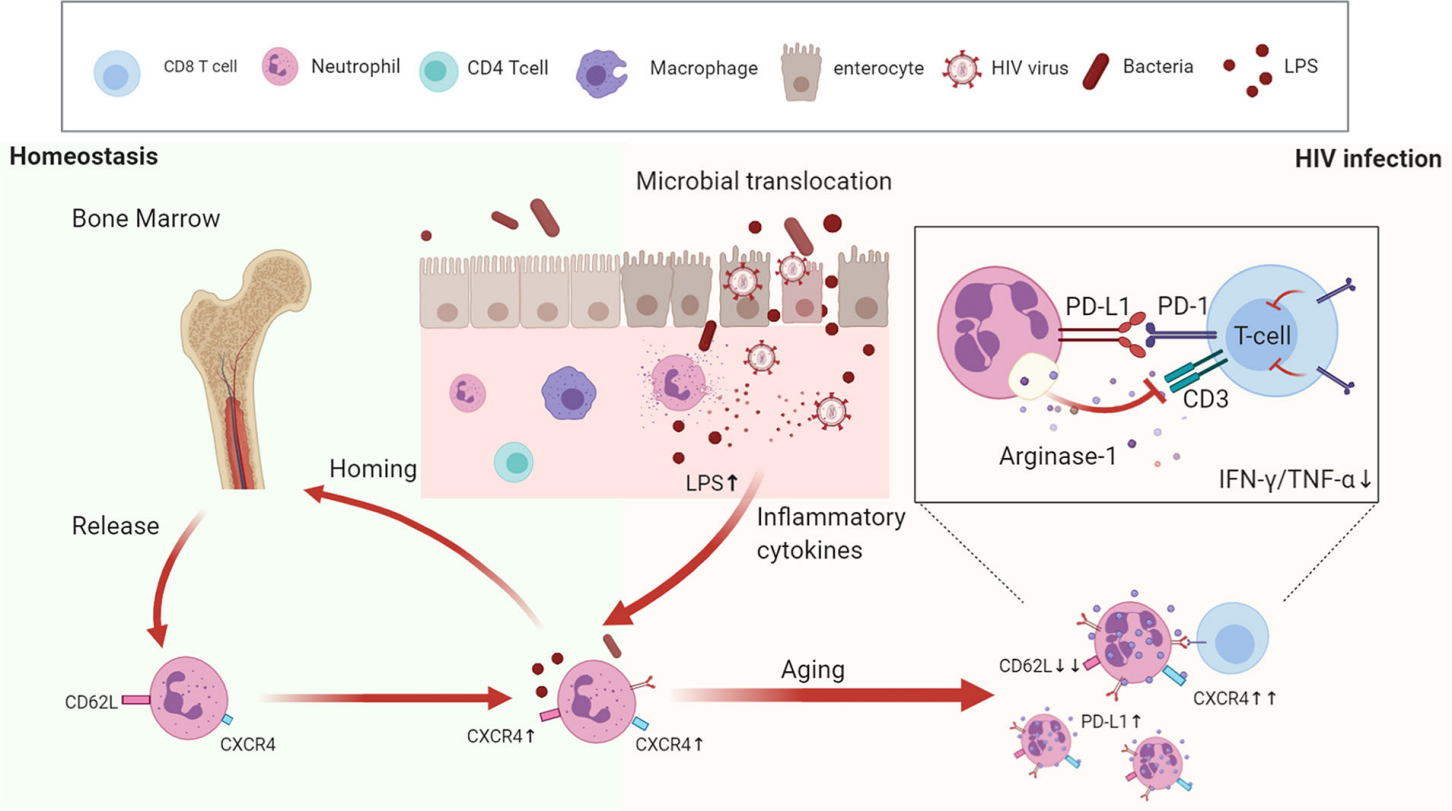
The aging neutrophil model in HIV-1-infected patients. Under normal physiological conditions, neutrophils undergo aging once they are released from the bone marrow, and then cleared by resident macrophages through CXCR4-CXCL12 receptors. During HIV-1 infection, the damaged intestinal barrier, accompanied by bacterial translocation, contribute to create a pro-inflammatory microenvironment, which promotes neutrophil aging. Aged neutrophils are characterized by multiple functional and phenotypic changes, which have stronger immunosuppressive effects on T cells through the increased expression of PD-L1 and arginase-1.

In summary, we reveal that during HIV-1 infection, excessive aging of neutrophils induces immunosuppression of T cells, detected by increased PD-L1 and arginase-1 expression, and LPS may be an important inducer for the aging process (**Figure 7**). Therefore, dampening the progress of neutrophil aging may provide a novel approach to recover T cell dysfunction in HIV-1-infected patients.

## Data Availability Statement

The datasets generated for this study are available on request to the corresponding authors.

## Ethics Statement

The studies involving human participants were reviewed and approved by the institutional review boards of Fifth Medical Center of Chinese PLA General Hospital. The patients/participants provided their written informed consent to participate in this study.

## Author Contributions

F-SW and RX conceived the study, wrote the manuscript, and constructed the figures with KL and H-HH. The clinical samples were contributed by H-HH. F-SW revised the manuscript and figures. C-BZ and J-HY performed flow cytometry. J-YZ, Y-MJ, CZ, J-WS, TY, X-YM, H-JL, and MS contributed to scientific planning. KL, W-JC, and M-JZ performed the laboratory work. Intellectual input was provided by all authors. All authors contributed to the article and approved the submitted version.

## Funding

The work was supported by grants from the National Science and Technology Fund (Major Project 2018ZX10302104--003), the National Natural Innovation Fund (Project 81721002), and the Peking University Clinical Scientist Program Special (BMU2019LCKXJ013).

## Conflict of Interest

The authors declare that the research was conducted in the absence of any commercial or financial relationships that could be construed as a potential conflict of interest.

## Publisher’s Note

All claims expressed in this article are solely those of the authors and do not necessarily represent those of their affiliated organizations, or those of the publisher, the editors and the reviewers. Any product that may be evaluated in this article, or claim that may be made by its manufacturer, is not guaranteed or endorsed by the publisher.
