# Supplementary figures and images for: Increased Neutrophil Aging Contributes to T Cell Immune Suppression by PD-L1 and Arginase-1 in HIV-1 Treatment Naïve Patients
Source: Front Immunol. 2021 Aug 18;12:670616. doi: 10.3389/fimmu.2021.670616 (PMC8416527; doi:10.3389/fimmu.2021.670616)

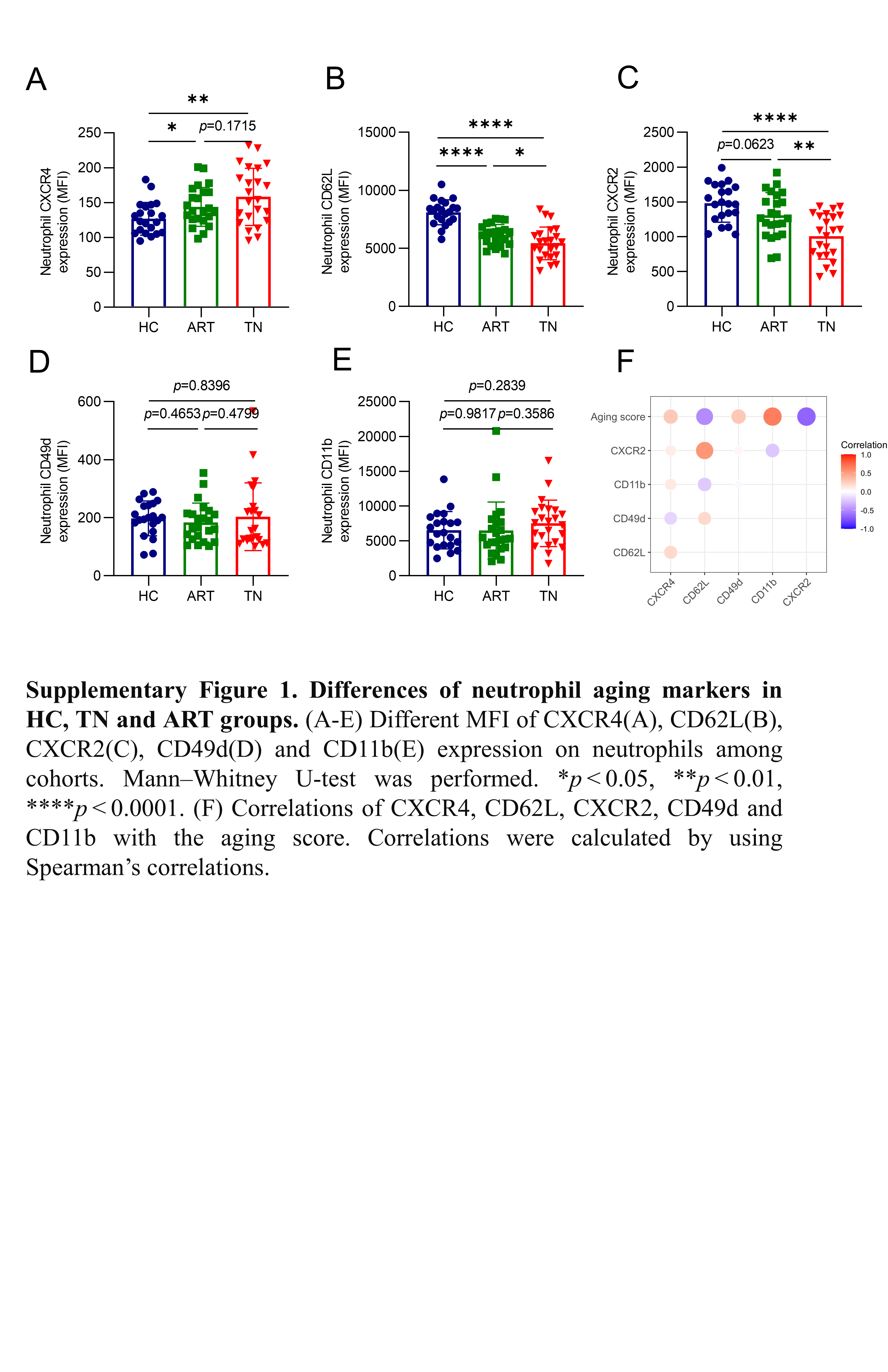

Supplement: Supplementary file 1 [file Image_1.tif]

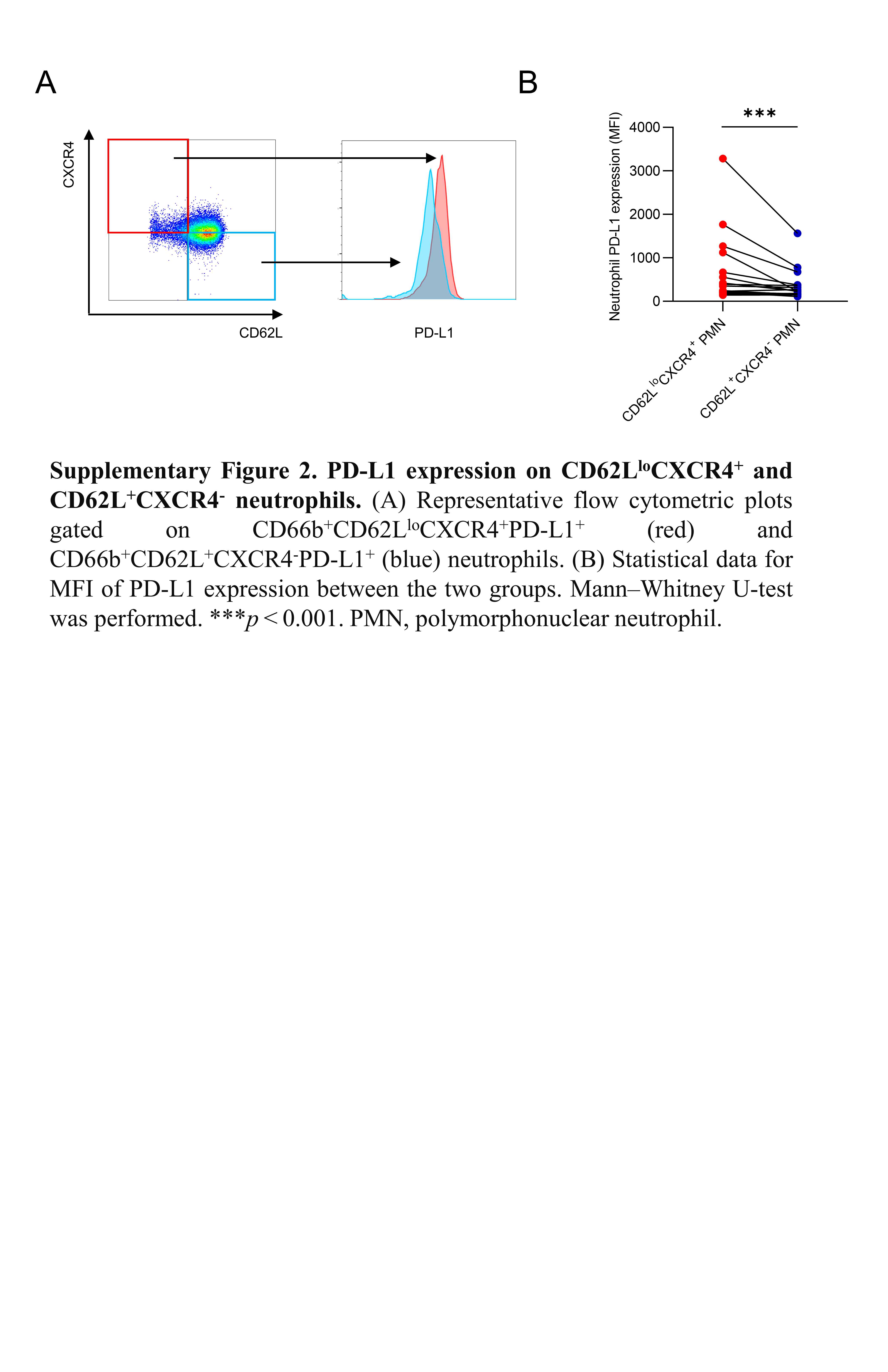

Supplement: Supplementary file 2 [file Image_2.tif]

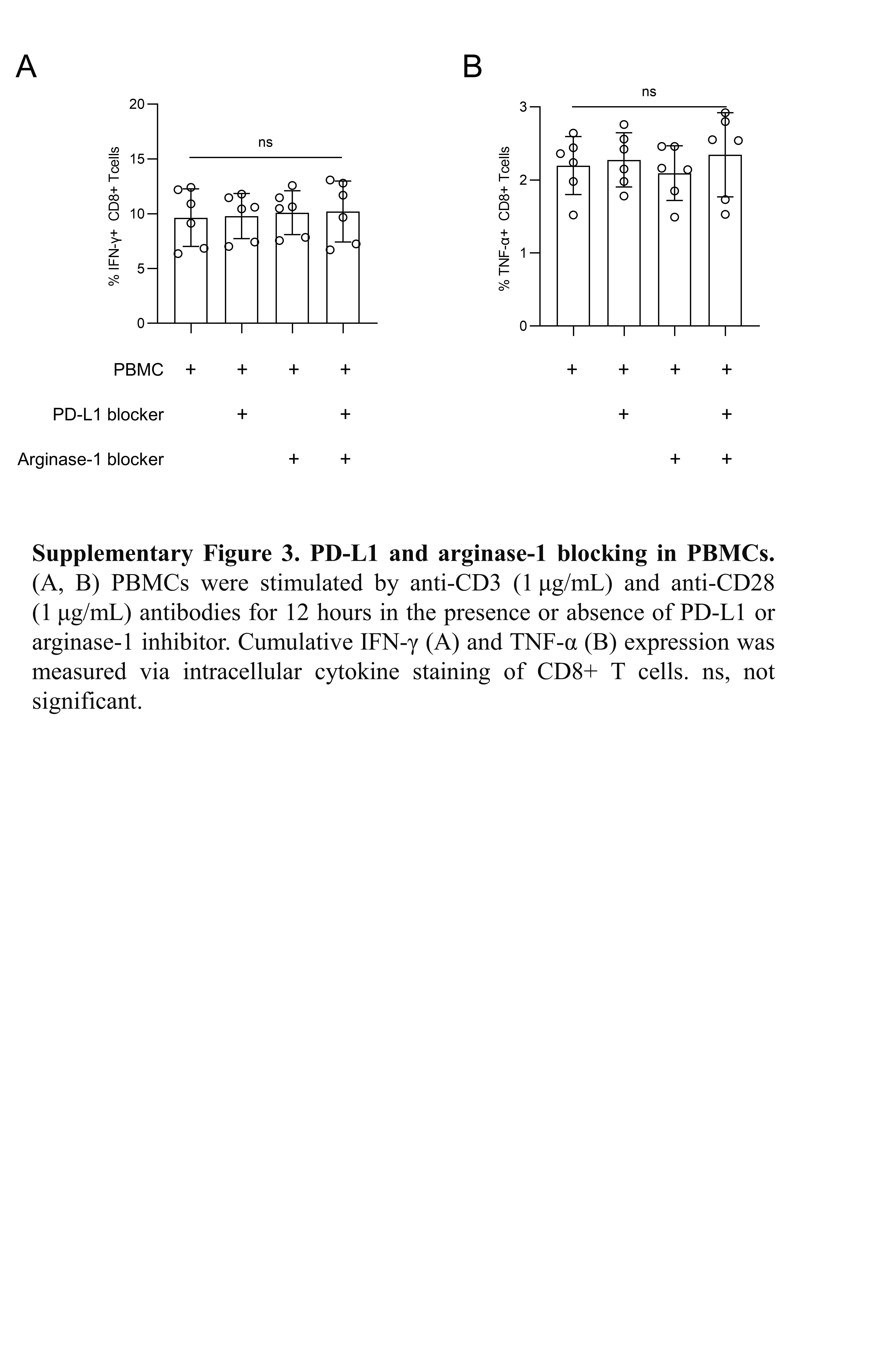

Supplement: Supplementary file 3 [file Image_3.tif]

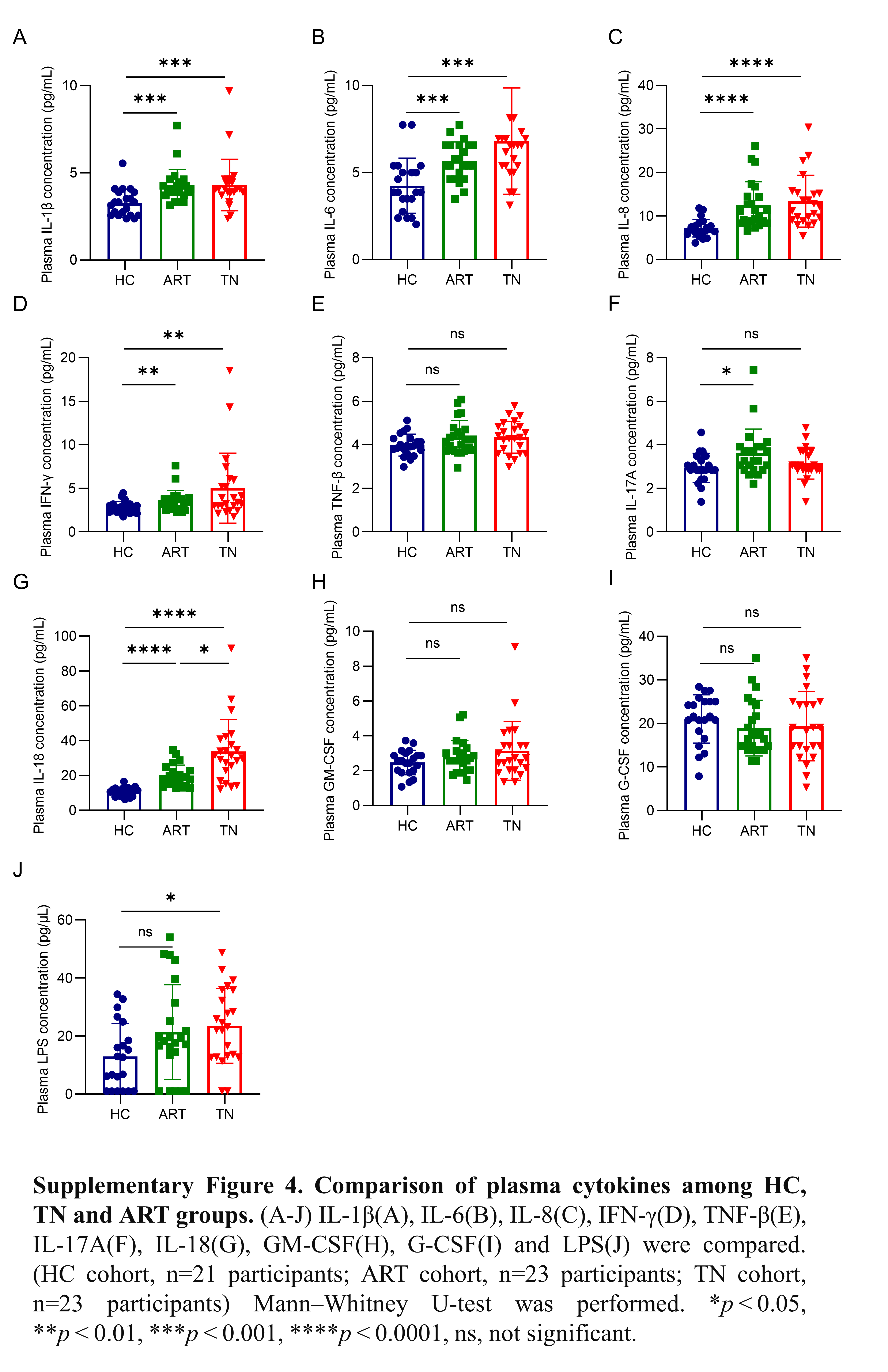

Supplement: Supplementary file 4 [file Image_4.tif]
